# Genome-Wide Association and Mechanistic Studies Indicate That Immune Response Contributes to Alzheimer’s Disease Development

**DOI:** 10.3389/fgene.2018.00410

**Published:** 2018-09-24

**Authors:** Changan Liu, Jacqueline Chyr, Weiling Zhao, Yungang Xu, Zhiwei Ji, Hua Tan, Claudio Soto, Xiaobo Zhou, for the Alzheimer’s Disease Neuroimaging Initiative

**Affiliations:** ^1^School of Biomedical Informatics, The University of Texas Health Science Center at Houston, Houston, TX, United States; ^2^Department of Neurology, The George and Cynthia W Mitchell Center for Alzheimer’s Disease and Other Brain Related Illnesses, McGovern Medical School, The University of Texas Health Science Center at Houston, Houston, TX, United States

**Keywords:** Alzheimer’s disease, GWAS, SNP, mechanism, transcription factor, binding affinity, regulation, immune disorders

## Abstract

Alzheimer’s disease (AD) is the most common cause of dementia. Although genome-wide association study (GWAS) have reported hundreds of single-nucleotide polymorphisms (SNPs) and genes linked to AD, the mechanisms about how these SNPs modulate the development of AD remain largely unknown. In this study, we performed GWAS for three traits in cerebrospinal fluid (CSF) and one clinical trait in the Alzheimer’s Disease Neuroimaging Initiative (ADNI) cohort. Our analysis identified five most significant AD related SNPs (FDR < 0.05) within or proximal to APOE, APOC1, and TOMM40. One of the SNPs was co-inherited with APOE allele 4, which is the most important genetic risk factor for AD. Three of the five SNPs were located in promoter or enhancer regions, and transcription factor (TF) binding affinity calculations showed dramatic changes (| Log2FC| > 2) of three TFs (PLAG1, RREB1, and ZBTB33) for two motifs containing SNPs rs2075650 and rs157580. In addition, our GWAS showed that both rs2075650 and rs157580 were significantly associated with the poliovirus receptor-related 2 (PVRL2) gene (FDR < 0.25), which is involved in spreading of herpes simplex virus (HSV). The altered regulation of PVRL2 may increase the susceptibility AD patients to HSV and other virus infections of the brain. Our work suggests that AD is a type of immune disorder driven by viral or microbial infections of the brain during aging.

## Introduction

Alzheimer’s disease (AD) is the most common neurodegenerative disorder. It is characterized by progressive memory loss and cognitive decline, cerebral accumulation of amyloid-β peptide (Aβ) in senile plaques and hyper-phosphorylated tau in neurofibrillary tangles (NFT) (Price and Sisodia, 1998; Mukaetova-Ladinska et al., 2015). Since AD is a complex and multifactorial disease, large datasets with multiple data types have been critical to identify its genetic risk factors (Harold et al., 2009). For several decades, only the allele 4 of Apolipoprotein E (APOE), which is present in about half of late-onset AD (LOAD) patients, has been convincingly demonstrated to affect risk for LOAD (Bertram et al., 2010).

Studies have shown that the levels of amyloid-β 1-42 peptide (Aβ_42_), total tau (T-tau), and tau phosphorylated at threonine 181 (p-tau) in cerebrospinal fluid (CSF) samples can be used as AD diagnostic biomarkers (Shaw et al., 2009; Hampel et al., 2010). AD patients show lower levels of CSF Aβ_42_ (Noguchi et al., 2005), which is negatively correlated with Aβ plaque counts in brain samples (Ikonomovic et al., 2008). The CSF levels of T-tau and p-tau are increased in AD patients (Price and Morris, 1999). Increased CSF T-tau levels are also found in stroke (Hesse et al., 2001) and traumatic brain injury (Ost et al., 2006). However, elevated CSF p-tau levels appear to be specific to AD (Buerger et al., 2006). The 13-item version of the Alzheimer’s Disease Assessment Scale-Cognitive subscale (ADAS13) was developed to measure memory and cognition for patients with mild to moderate AD (Podhorna et al., 2016). It’s one of the most frequently applied tests in experimental studies and clinical trials for new drugs and other interventions. A normal ADAS13 score for a person who does not have AD is 5 (Graham et al., 2004), while 31.2 is the average score for those who have been diagnosed with AD or mild cognitive impairment (The Alzheimer’s Disease Neuroimaging Initiative et al., 2015).

**FIGURE 1:**
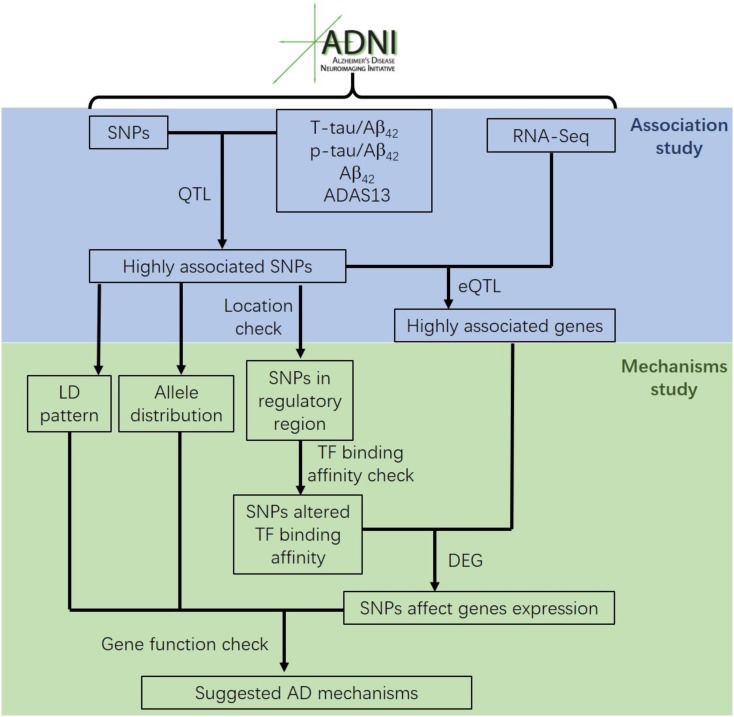
The flow chart of the analysis conducted in this study.

Most genetic association studies analyze at a single marker level or focus on detecting risk factors for AD, but ignore the mechanisms and functions associated with what they find. In our study, we conducted quantitative trait locus (QTL) analysis of Aβ_42_, T-tau/Aβ_42_ ratio, p-tau/Aβ_42_ ratio and ADAS13 as quantitative traits on SNPs to identify significantly AD-associated SNPs. We then performed expression quantitative trait loci (eQTL) analysis for these AD-associated SNPs to locate the related genes. After that, we computed the linkage disequilibrium (LD) pattern, allele distributions and transcription factor motifs binding affinity for SNPs in regulatory regions (promoter regions or enhancer regions) to study how they modulate the target genes (**Figure 1**). Overall, our analysis may contribute to understand the mechanism and etiology of AD.

## Materials and Methods

Data used in this study were obtained from the Alzheimer’s Disease Neuroimaging Initiative (ADNI) database (adni.loni.usc.edu). ADNI was launched in 2003 by the National Institute on Aging (NIA), the National Institute of Biomedical Imaging and Bioengineering (NIBIB), the Food and Drug Administration (FDA), and by private pharmaceutical companies and non-profit organizations, as a public-private partnership. The principal investigator of ADNI is Michael W. Weiner, MD. The primary goal of ADNI has been to test whether serial magnetic resonance imaging (MRI), positron emission tomography (PET), biological markers, and clinical and neuropsychological assessment can be combined together to measure the progression of AD.

The first phase of ADNI (ADNI-1) launched in 2004 included 400 subjects diagnosed with late mild cognitive impairment (LMCI), 200 subjects with AD, and 200 elderly cognitively normal (CN) subjects. ADNI was extended in 2009 during the ADNI-GO phase, which assessed the existing ADNI-1 cohort along with 200 new participants with early mild cognitive impairment (EMCI). In 2011, assessing participants from ADNI-1/ADNI-GO phases, ADNI-2 began with new subject groups: 150 CN, 100 EMCI, 150 LMCI and 150 AD.

We were granted permission to obtain data from the ADNI cohort (www.adni-info.org) for performing the analysis described in this paper.

### Subjects

We analyzed the CSF Aβ_42_, T-tau and p-tau levels, ADAS13 scores, quality-controlled SNP data and gene expression data for 812 ADNI subjects, including 281 CN, 235 EMCI, 249 LMCI and 47 AD cases.

### Genotyping Data

The SNP data of ADNI-1, ADNI-GO, and ADNI-2 cohorts were collected from either the Illumina 2.5-M array or the Illumina OmniQuad array (Saykin et al., 2010). The SNPs shown in both arrays were used for the following analysis.

Quality control (QC) analysis was conducted by using R package snpStats (Clayton, 2012) in R software (R Core Team, 2013). In the QC, we excluded any SNPs that did not meet any of the following criteria: (1) SNPs on chromosome 1-22; (2) call rate per SNP > 95%; (3) minor allele frequency (MAF) > 5%; (4) Hardy-Weinberg equilibrium (HWE) test of *p*-value > 10^-6^ (absolute value of *z*-score < 4.753424). After QC analysis, 575353 SNPs remained for the subsequent analysis.

### CSF Biomarkers and ADAS13

The CSF levels of Aβ_42_, T-tau and p-tau were determined using the fully automated Roche Elecsys immunoassay platform (Seibyl et al., 2017). The ADAS13 was conducted by a certified ADAS13 rater. ADAS13 scores were automatically calculated on the electronic case report form based on item level data entered.

### Gene Expression

Gene expression profiles of peripheral blood samples from ADNI participants were performed at Bristol-Myers Squibb (BMS) laboratories. The Affymetrix Human Genome U219 Array (www.affymetrix.com) was used for expression profiling, which contains 530467 probes for 49293 transcripts. Raw expression values obtained directly from CEL files were pre-processed using the RMA (Robust Multi-chip Average) normalization method (Vawter et al., 2004).

We also used the dataset GSE28146 (Blalock et al., 2011) in Gene Expression Omnibus [GEO (Barrett et al., 2012)]. The gene expression data was collected from laser-captured hippocampus tissue gray matter from formalin fixed, paraffin embedded specimens by Affymetrix Human Genome U133 Plus 2.0 Array. The dataset includes eight controls and seven cases of severe AD.

### Regulatory Information for SNP Location

The regulatory region information (promoter, enhancer and TF binding regions) of the identified AD-associated SNPs was obtained from Genome Browser (Kent et al., 2002) and HaploReg (Ward and Kellis, 2012). We used TF binding motifs data in Motif browser (Kheradpour and Kellis, 2014) and R package MotifDb (Shannon, 2014) with motifs in JASPAR (Khan et al., 2018), SwissRegulon (Pachkov et al., 2012) and other databases, for the computation of binding affinity.

### Analysis

To determine the SNPs associated with Aβ_42_ level, T-tau/Aβ_42_ ratio, p-tau/Aβ_42_ ratio in CSF and ADAS13 score, we performed QTL analysis using the R package MatrixEQTL (Shabalin, 2012). In this analysis, age, gender (1 for male and 2 for female) and diagnosis (1 for CN, 2 for EMCI, 3 for LMCI and 4 for AD) at baseline were considered as covariates. Manhattan plots of QTL results were generated using the R package qqman (Turner, 2014). Following analysis was restricted to the associated SNPs on the chromosome 19 (for APOE on chromosome 19) with the false discovery rate (FDR) < 0.05. eQTL analysis was also conducted using the R package MatrixEQTL for the filtered SNPs, with the same covariates as QTL. The cis-eQTL results (local, distance < 1 Mb) with FDR < 0.25 were considered as significant. The LD pattern was plotted using the R package LDheatmap (Shin et al., 2006).

TF motifs binding affinity were calculated using the R package PWMEnrich for the SNPs in the regulatory regions. We used the regions located +/- 20 base pair (bp) of the SNPs as the DNA strings to perform the computation of binding affinity. To determine the changes in binding affinity, we calculated the log2 fold change (Log2FC) of binding affinity for major alleles against the binding affinity for minor alleles. TF motifs with binding affinity of both sequences with major allele and minor allele less than 1 (unlikely to bind on the sequences) were excluded for the following analysis. Remaining TF motifs and related SNPs with absolute value of Log2FC greater than 2 (|Log2FC| > 2) were used for the functional and mechanism analysis.

Gene differential expression between CN group and AD group was conducted by the R package limma (Ji et al., 2014; Ritchie et al., 2015). We checked the PVRL2 gene expression for both ADNI and dataset GSE28146 from GEO.

## Results

**FIGURE 2:**
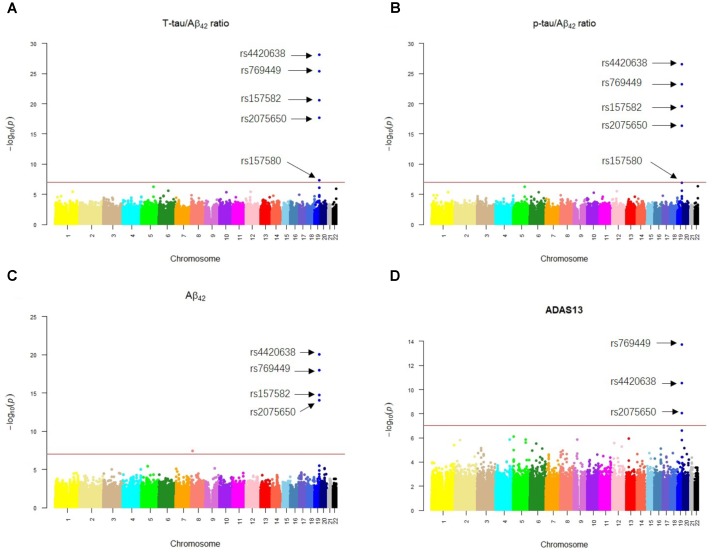
The Manhattan plots of the observed –log_10_ (*p*-value) for the results of QTL analysis for **(A)** T-tau/Aβ_42_ ratio, **(B)** p-tau/Aβ_42_ ratio, **(C)** Aβ_42_ concentration, and **(D)** ADAS13 scores. SNPs with FDR < 0.05 were labeled on each plot. Redline stood for *p*-value = 10^-7^.

Our results of QTL analysis for Aβ_42_, T-tau/Aβ_42_ ratio, p-tau/Aβ_42_ ratio and ADAS13 (**Supplementary Tables S1**–**S4**) were illustrated in the Manhattan plots (**Figure 2**), showing that almost all SNPs significantly associated with AD (*p*-value < 10^-7^) are located at the chromosome 19 which contains APOE gene. **Table 1** shows the SNPs with FDR < 0.05 from the QTL analysis of Aβ_42_, T-tau/Aβ_42_ ratio, p-tau/Aβ_42_ ratio and ADAS13, respectively. A total of five SNPs were identified according to the threshold FDR < 0.05, and they were also reported by previous studies (Li et al., 2017). Detected SNPs rs2075650, rs157580 and rs157582 are located within gene TOMM40. SNP rs769449 locates within gene APOE and rs4420638 is proximal to the downstream of gene APOC1. SNP rs4420638 is the most significant AD-associated SNP in our QTL analysis for Aβ_42_ (FDR = 5.47E-15), T-tau/Aβ_42_ ratio (FDR = 4.28E-23), and p-tau/Aβ_42_ ratio (FDR = 1.78E-21; **Figure 2** and **Table 1**). It is also the second most significant AD-associated SNP in our QTL analysis for ADAS13 (FDR = 8.73E-06). These imply that rs4420638 is the most relevant SNP to AD in ADNI according to our QTL analysis. The significant SNPs identified based on the T-tau/Aβ_42_ ratio and p-tau/Aβ_42_ ratio are identical due to the number and the order of SNPs (**Figure 2** and **Table 1**). SNPs rs4420638, rs769449, and rs2075650 were identified to be significant for all of the four traits and ranked in the top three (**Figure 2** and **Table 1**).

**Table 1:** Results of QTL analysis.

SNP	Gene	*p*-value	FDR
**T-tau/Aβ_42_**		
rs4420638	APOC1^∗^	7.43E-29	4.28E-23
rs769449	APOE	3.95E-26	1.14E-20
rs157582	TOMM40	2.69E-21	5.16E-16
rs2075650	TOMM40	2.03E-18	2.92E-13
rs157580	TOMM40	4.61E-08	0.0053
**p-tau/Aβ_42_**		
rs4420638	APOC1^∗^	3.09E-27	1.78E-21
rs769449	APOE	5.54E-24	1.59E-18
rs157582	TOMM40	2.6E-20	4.99E-15
rs2075650	TOMM40	4.63E-17	6.66E-12
rs157580	TOMM40	1.16E-07	0.013402
**Aβ_42_**		
rs4420638	APOC1^∗^	9.51E-21	5.47E-15
rs769449	APOE	1.11E-18	3.18E-13
rs157582	TOMM40	1.72E-15	3.29E-10
rs2075650	TOMM40	8.34E-15	1.2E-09
**ADAS13**		
rs769449	APOE	1.88E-14	1.08E-08
rs4420638	APOC1^∗^	3.03E-11	8.73E-06
rs2075650	TOMM40	8.89E-09	0.001706


*Only QTL results with FDR < 0.05 are shown. If the SNP is located within a gene, the gene is listed, otherwise the nearest gene is listed. ^∗^Nearest gene proximal to the SNP. Without this symbol means the SNP is located within the corresponding gene.*

We then performed eQTL analysis for the five identified SNPs (**Supplementary Table S5**). **Table 2** shows the cis-eQTL results with FDR < 0.25. In order to include more SNPs and genes for the following mechanistic analysis, we set FDR < 0.25 as the threshold instead of the more classic cutoff FDR < 0.05 for our eQTL results. For we want to find the mechanism of AD, here we just focused on the cis-eQTL results and did not consider the trans-eQTL results. SNPs rs769449, rs2075650 and rs157580 were associated with PVRL2 gene (FDR = 0.19035, 0.0367, and 0.08866, respectively), while rs4420638 was associated with SYMPK gene (FDR = 0.22, **Table 2**). For three SNPs showed significant association with PVRL2 gene, we studied its function in the following. In addition to PVRL2 gene, rs2075650 was also associated with HIF3A gene (FDR = 0.19035, **Table 2**). There were no genes significantly associated with rs157582 according to our cis-eQTL results.

**Table 2:** Results of eQTL analysis for the five SNPs closely related to AD.

SNP	Probe ID	Gene	Statistic	*p*-value	FDR	Beta
rs4420638	11749529_a_at	SYMPK	2.88592	0.00402	0.22	0.05316
rs769449	11719528_at	PVRL2	2.97703	0.00301	0.19035	0.23487
rs2075650	11719528_at	PVRL2	3.79544	0.00016	0.0367	0.28469
	11722674_x_at	HIF3A	-3.122	0.00187	0.19035	-0.0358
rs157580	11718065_a_at	PVRL2	-3.4756	0.00054	0.08866	-0.2368


*Only eQTL results with FDR < 0.25 are listed.*

We calculated allele distributions and minor allele frequencies (MAF) of the five identified SNPs for the four diagnosis groups (**Table 3**) to study their different features among different groups. Except for rs157580, MAFs were increased along with the progression of AD. For rs4420638, rs769449, rs2075650, and rs157582, the percentage of subjects carrying two minor alleles was greater in the AD group than that in the other three diagnosis groups. These genetic features of AD group highlight the contribution of the genotype to the development of AD.

**Table 3:** Alleles distribution of the five AD-associated SNPs in four diagnostic groups.

	CN (281)	EMCI (235)	LMCI (249)	AD (47)
**rs4420638 (A/G)**				
AA	180 (64.06%)	139 (59.15%)	115 (46.18%)	15 (31.91%)
AG	91 (32.38%)	75 (31.91%)	104 (41.77%)	23 (48.94%)
GG	10 (3.56%)	21 (8.94%)	30 (12.05%)	9 (19.15%)
MAF	19.75%	24.89%	32.93%	43.62%
**rs769449 (G/A)**				
GG	228 (81.14%)	164 (69.79%)	145 (58.23%)	20 (42.55%)
GA	49 (17.44%)	63 (26.81%)	87 (34.94%)	22 (46.81%)
AA	4 (1.42%)	8 (3.40%)	17 (6.83%)	5 (10.64%)
MAF	10.14%	16.81%	24.30%	34.04%
**rs2075650 (A/G)**				
AA	204 (72.60%)	154 (65.53%)	140 (56.23%)	21 (44.68%)
AG	71 (25.27%)	70 (29.79%)	95 (38.15%)	22 (46.81%)
GG	6 (2.13%)	11 (4.68%)	14 (5.62%)	4 (8.51%)
MAF	14.77%	19.57%	24.70%	31.91%
**rs157582 (C/T)**				
CC	158 (56.23%)	123 (52.34%)	110 (44.18%)	17 (36.17%)
CT	109 (38.79%)	90 (38.30%)	110 (44.18%)	23 (48.94%)
TT	14 (4.98%)	22 (9.36%)	29 (11.64%)	7 (14.89%)
MAF	24.38%	28.51%	33.73%	39.36%
**rs157580 (A/G)**				
AA	104 (37.01%)	105 (44.68%)	115 (46.18%)	19 (40.43%)
AG	136 (48.40%)	100 (42.55%)	109 (43.78%)	24 (51.06%)
GG	41 (14.59%)	30 (12.77%)	25 (10.04%)	4 (8.51%)
MAF	38.79%	34.04%	31.93%	34.04%


*(^∗^/^∗^) The first allele is major allele while the second one is minor allele.*

**FIGURE 3:**
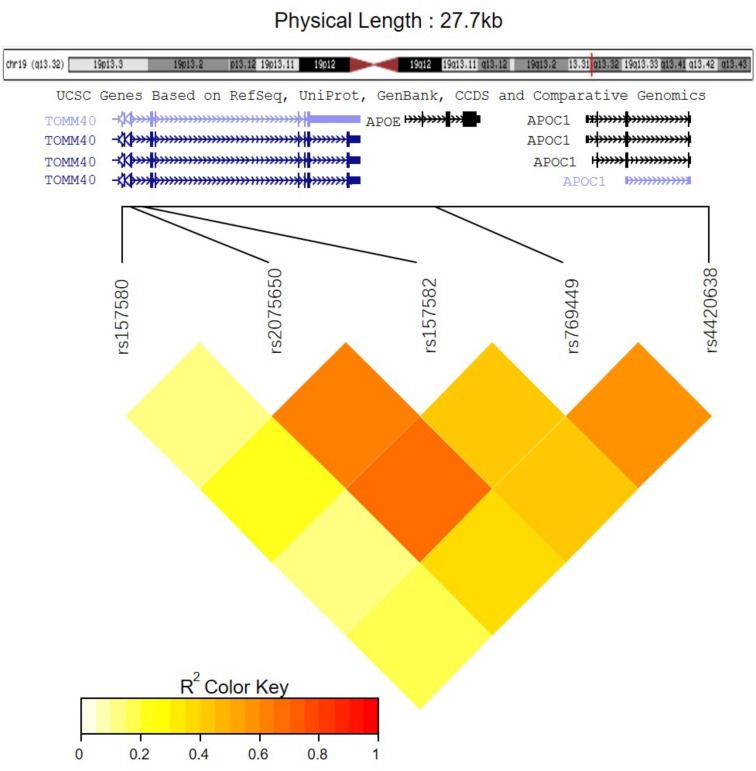
The LD pattern for the five significantly associated SNPs.

**FIGURE 4:**
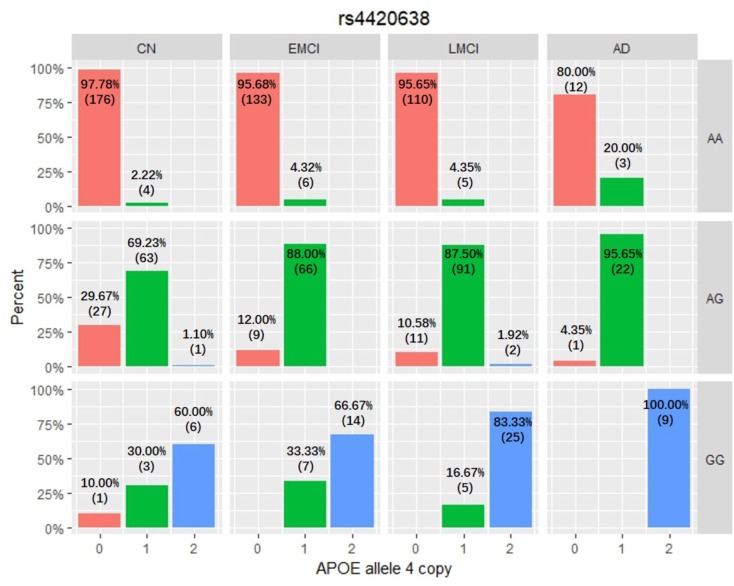
The relationship between APOE allele 4 copy numbers and rs4420638 alleles in four diagnostic groups.

Among these five identified SNPs, rs2075650 shows strong LD with rs769449 (*D*′ = 0.91, *r*^2^ = 0.69; **Figure 3**) and rs157582 (*D*′ = 1.00, *r*^2^ = 0.61; **Figure 3**). The strong LD between rs2075650 and rs157582 may be due to that both of them locate within gene TOMM40 closely. Moreover, rs4420638 shows strong LD with rs769449 (*D*′ = 0.97, *r*^2^ = 0.56; **Figure 3**). Besides the LD pattern for these five SNPs, we checked that SNP rs4420638 was in strong LD with rs429358 (*D*′ = 0.94, *r*^2^ = 0.65; by HaploReg), which contributes to define the allele types of APOE gene. We explored the relationship between rs4420638 alleles and the APOE allele 4 copy numbers (**Figure 4**). Our results showed that minor allele G of rs4420638 was highly correlated with APOE allele 4 copy numbers. Most subjects carrying one or two G alleles have one or two copies of APOE allele 4 among all the four diagnosis groups, respectively. This situation was much more obvious for AD group. All AD patients with two G alleles had two copies of APOE allele 4. All but one (95.65%) AD subjects carrying one allele G had just one APOE allele 4.

**FIGURE 5:**
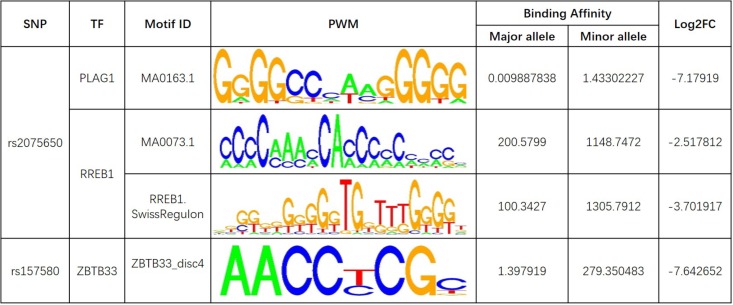
The transcription factor binding affinity for AD related SNPs in regulatory regions. Only binding affinity of TF motifs that are largely altered (|Log2FC| > 2) by the SNP genotypes are listed. PWM, position weight matrix. Log2FC, Log2 fold change for the binding affinity of sequence with major allele against the binding affinity of sequence with minor allele.

Further analysis shows that rs769449, rs2075650, and rs157580 are located in promoter and enhancer regions for multiple types of brain tissues and these SNPs are also located in TF binding regions (annotations from Genome Browser and HaploReg). We calculated the corresponding TF motifs binding affinity for these three SNPs (**Supplementary Table S6**). SNP rs2075650 and rs157580 significantly altered the binding affinity (|Log2FC| > 2) of four TF motifs (**Figure 5**). SNP rs2075650 altered the binding affinity of TFs PLAG1 (Log2FC = -7.17919) and RREB1 (Log2FC = -2.517812 for motif MA0073.1 and Log2FC = -3.701917 for motif RREB1.SwissRegulon) while rs157580 only altered the binding affinity of TF ZBTB33 (Log2FC = -7.642652). All of the four TF motifs had higher binding affinity scores to the sequences containing the minor allele of the related SNPs, indicating that the regulatory function of these TFs may be enhanced for the subjects carrying minor allele of rs2075650 and rs157580. Through altering the regulatory function of these TFs, these SNPs can affect their target genes.

## Discussion

APOE has three different alleles (allele 2, 3, and 4) that are defined by two SNPs: rs429358 (T/C, T as major allele) and rs7412 (C/T, C as major allele). APOE allele 2 [(T;T) for (rs429358 and rs7412)] is relatively rare and considered to be protective against AD. The most common allele is the APOE allele 3 (T;C) which is believed to have no effect on AD (Farrer, 1997). APOE allele 4 (C;C) increases the risk for developing AD (Liu et al., 2013). Due to the diploid nature of the human genome, a normal person can have zero, one, or two copies of APOE allele 4. Carrying one copy of APOE allele 4 can increase the risk of AD by 2 to 3 times, while carrying two such copies have a 25-fold increased risk for developing AD compared to people with two APOE allele 3 (Michaelson, 2014). SNP rs4420638 is the most significant AD-associated SNP identified in our study (**Figure 2** and **Table 1**). Its minor allele G occurs almost simultaneously with APOE allele 4, especially in AD patients (**Figure 4**). More importantly, increased MAF for this SNP was observed in the AD patients (**Table 3**). These results suggest that rs4420638 (allele G) is co-inherited with APOE allele 4, which is supported by the previous studies (Li et al., 2008).

Pleomorphic adenoma gene 1 (PLAG1), encoding a zinc finger protein with 2 putative nuclear localization signals, has been shown to be consistently rearranged in pleomorphic adenomas of the salivary glands. As a transcription factor, its activation leads to the up-regulation of target genes (Van Dyck et al., 2007). Ras-Responsive Element-Binding Protein 1 is a zinc finger transcription factor encoded by RREB1 gene. It binds to RAS-responsive elements (RREs) of gene promoters and potentiates the transcriptional activity of Neurogenic differentiation 1 gene (NEUROD1) and other genes (Melani et al., 2008). SNP rs2075650 altered the binding affinity for some motifs of PLAG1 and RREB1, where minor allele G increased the binding affinity of these motifs for both TFs (**Figure 5**). Moreover, AD patients had the highest MAF for this SNP among all the four diagnosis groups (**Table 3**), implying increased function and regulation of these two TFs and increased activation of their target genes in the subjects of AD group. Zinc finger and BTB domain-containing protein 33 (ZBTB33) encodes a transcriptional regulator with bimodal DNA-binding specificity. It recruits the N-CoR repressor complex to promote histone deacetylation and the formation of repressive chromatin structures in target gene promoters. In addition, it may contribute to the repression of target genes (Daniel and Reynolds, 1999). By our analysis, ZBTB33 is more likely to bind to the string with minor allele G of rs157580 (**Figure 5**), compared with the string containing major allele A. The MAF of rs157580 in AD group was less than that in the CN group (**Table 3**), suggesting that the regulatory function of ZBTB33 in AD patients is weaker than that in the healthy people. Our eQTL analysis indicated that both rs2075650 and rs157580 were significantly associated with the PVRL2 gene (**Table 2**). PVRL2 appears to be the common target gene of the transcription factor PLAG1, RREB1, and ZBTB33. The altered binding affinity of these TFs in AD patients may potentiate the transcriptional activity of PVRL2. More importantly, PVRL2 was found to be upregulated in AD patients compared to CN in ADNI and another dataset (**Table 4**).

**Table 4:** PVRL2 gene differential expression for AD group against CN group in ADNI and GSE28146.

Data	Probe ID	Log2FC	AveExpr	*t*	*p*-value	adj.p.val	*B*
ADNI	11718065_a_at	0.3711096	4.0623	1.817783	0.07008629	0.5363667	-4.129824
GSE28146	225418_at	0.304203702	4.007755	2.09615834	0.04408898	0.1763559	-3.76633


*AveExpr, average log2-expression; t, t-statistic; adj.p.val, adjusted *p*-value; B, log-odds that the gene is differentially expressed.*

Poliovirus receptor-related 2 (PVRL2) gene, also known as nectin cell adhesion molecule 2 (NECTIN2) or herpesvirus entry mediator B (HVEB), is a human plasma membrane glycoprotein (Lopez et al., 1998). PVRL2 gene is located in close vicinity of the APOE locus on the chromosome 19. It is expressed in multiple types of cell and tissues, including neurons. It belongs to the plasma membrane components of adherens junctions (Takai, 2003). More importantly, it mediates the entry of herpes simplex virus (HSV) (Warner et al., 1998). Dysregulation for PVRL2 may have impact on the susceptibility of individuals to HSV infection of brain by affecting virus entry to cells and intercellular virus spreading (Spear, 2004). There is evidence showing that HSV infection in AD brains was observed (Itzhaki and Wozniak, 2008). A recent study reported that Aβ peptide might be involved in resistance to microbial infection in mouse and worm models of AD and acted as a defense molecule of the innate immunity (Kumar et al., 2016), which is compatible with the viral association with AD etiology and pathology. The accumulation of Aβ plaque deposits may be a consequence of the over-production of Aβ peptide during viral infection of the brain (Porcellini et al., 2010). Additionally, some previous studies showed that HSV could contribute to the development of Aβ plaques directly (Piacentini et al., 2011). What’s more, reactivated HSV1 can directly induce inflammatory damage, which may lead to an increase on the formation of Aβ and tau pathology (Wozniak et al., 2007). Last but the least, a new study of late-onset AD-associated virome found the evidence that links the activity of specific viral species (includes HSV1) with molecular, genetic, clinical and neuropathological aspects of AD (Readhead et al., 2018).

**FIGURE 6:**
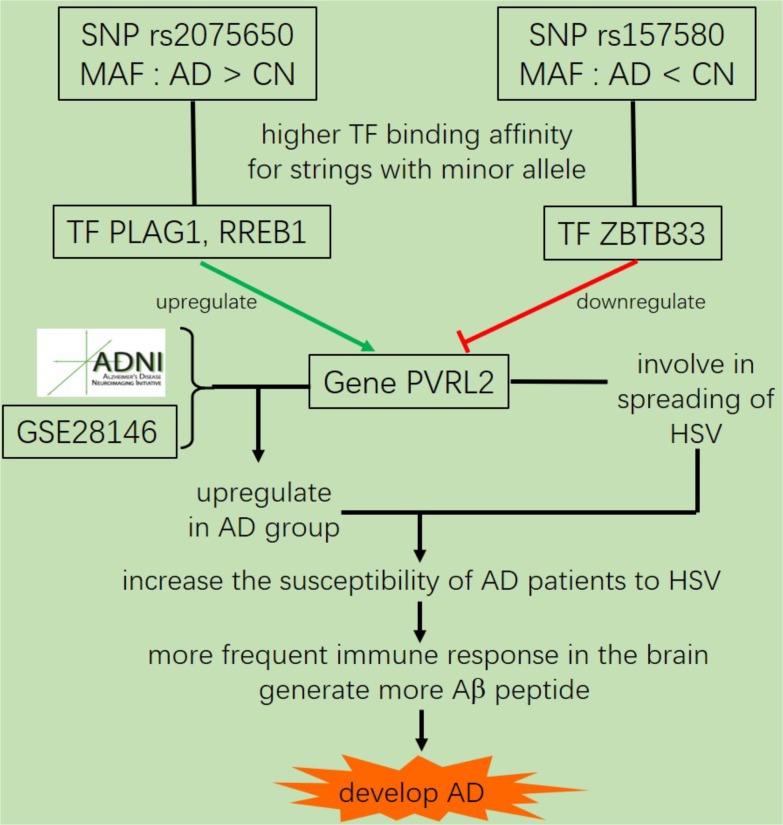
The suggested AD mechanism according to our analysis.

In summary, we identified five SNPs that were highly associated with AD by QTL analysis for multiple traits. Among them, the allele G of rs4420638 is co-inherited with the APOE allele 4. In addition, our eQTL analysis indicated that both rs2075650 and rs157580 were significantly associated with the PVRL2 gene. The transcription factors PLAG1, RREB1 and ZBTB33 had higher binding affinities to the motifs containing minor allele of the rs2075650 and rs157580, indicating an enhanced regulatory function of these TFs in the subjects with such minor alleles. The altered binding affinity of these TFs to these SNP regions increased the expression of PVRL2 in AD patients as compared to CN. Increased expression of PVRL2 may also increase the patients’ susceptibility to HSV and other viral infections of the brain. Overtime, the more frequent immune activation against infections may result in progressive neurodegeneration (**Figure 6**). Our findings suggest that AD is a type of immune disorder driven by virus or microbial infections of the brain. For microglia are the brain-resident immune cells and TREM2 gene plays a crucial role in microglia function (Keren-Shaul et al., 2017; Kober and Brett, 2017), we will study how they affect the progression of AD for further research.

## Alzheimer’s Disease Neuroimaging Initiative

Data used in preparation of this article were obtained from the Alzheimer’s Disease Neuroimaging Initiative (ADNI) database (adni.loni.usc.edu). As such, the investigators within the ADNI contributed to the design and implementation of ADNI and/or provided data but did not participate in analysis or writing of this report. A complete listing of ADNI investigators can be found at: http://adni.loni.usc.edu/wpcontent/uploads/how_to_apply/ADNI_Acknowledgement_List.pdf.

## Author Contributions

CL analyzed the data and wrote the manuscript. JC, CS, and WZ helped with the interpretation of the results and writing of the manuscript. YX, ZJ, and HT helped with the study design and statistical analysis. XZ oversaw the overall research plan, the study design and statistical analysis. All authors read and approved the manuscript.

## Conflict of Interest Statement

The authors declare that the research was conducted in the absence of any commercial or financial relationships that could be construed as a potential conflict of interest.
