# Supplementary material for: Genome-Wide Association and Mechanistic Studies Indicate That Immune Response Contributes to Alzheimer’s Disease Development
Source: Front Genet. 2018 Sep 24;9:410. doi: 10.3389/fgene.2018.00410 (PMC6166008; doi:10.3389/fgene.2018.00410)
Supplement: Supplementary file 7 [file Data_Sheet_1.DOCX]

Supplementary Material

Genome-Wide Association and Mechanistic Studies Indicate that Immune Response Contributes to Alzheimer’s Disease Development

Changan Liu, Jacqueline Chyr, Weiling Zhao, Yungang Xu, Zhiwei Ji, Hua Tan, Claudio Soto, Xiaobo Zhou*, for the Alzheimer’s Disease Neuroimaging Initiative

*** Correspondence:** Xiaobo Zhou: [Xiaobo.Zhou@uth.tmc.edu](mailto:Xiaobo.Zhou@uth.tmc.edu)

# Supplementary Tables

**Table S1.** Full results of QTL analysis for T-tau/Aβ_42_ ratio.

**Table S2.** Full results of QTL analysis for p-tau/Aβ_42_ ratio.

**Table S3.** Full results of QTL analysis for Aβ_42_ level.

**Table S4.** Full results of QTL analysis for ADAS13 scores.

**Table S5.** Full results of cis-eQTL analysis for the five SNPs closely related to AD.

**Table S6.** Full results of transcription factor binding affinity for AD related SNPs rs769449, rs2075650 and rs157580.
